# Comparative effectiveness of second generation long-acting injectable antipsychotics based on nationwide database research in Hungary

**DOI:** 10.1371/journal.pone.0218071

**Published:** 2019-06-13

**Authors:** P. Takács, P. Czobor, L. Fehér, J. Gimesi-Országh, P. Fadgyas-Freyler, M. Bacskai, P. Rakonczai, A. Borsi, R. Hegyi, T. Németh, J. Sermon, I. Bitter

**Affiliations:** 1 Janssen Global Commercial Strategy Organization, Budapest, Hungary; 2 Department of Psychiatry and Psychotherapy, Semmelweis University, Budapest, Hungary; 3 Janssen-Cilag Hungary Ltd., Budapest, Hungary; 4 Healthware Ltd., Budapest, Hungary; 5 National Health Insurance Fund Administration, Budapest, Hungary; 6 Janssen Pharmaceutica N.V., Beerse, Belgium; University of Toronto, CANADA

## Abstract

**Background:**

Schizophrenia is a severe condition that affects approximately 1% of the population. Certain elements of antipsychotic treatment can only be examined in large population, thus the need for population-based real-world analyses has been increasing.

**Patients and methods:**

Hungarian National Health Fund database includes all healthcare data of the population of Hungary. All patients diagnosed with schizophrenia between 01.01.2006 and 31.12.2015 were included in the study. We analyzed all patients with newly initiated second-generation antipsychotic during the inclusion period (01.01.2012–31.12.2013). Patients were followed for 2 years. All-cause treatment discontinuation served as the primary outcome of the study. Patients with newly initiated long-acting injectable treatments were further investigated in stratified analyses based on their previous treatment.

**Results:**

106,624 patients had schizophrenia diagnosis during the study period. 12,232 patients met the inclusion criteria for newly initiating second-generation antipsychotic during the inclusion period. The proportion of patients still on treatment after 1 year for oral treatments varied between 17% (oral risperidone) and 31% (oral olanzapine) while the analogous data for long acting injectables were between 32% (risperidone long acting) and 64% (paliperidone long acting one monthly). The 2-year data were similarly in favor of long-actings. Median time to discontinuation in the oral group varied between 57 days (clozapine) and 121 days (olanzapine). The median time to discontinuation for long-actings was significantly longer: between 176 and 287 days; in case of paliperidone long acting, median was not reached during the observation period. Patients receiving long-acting treatment switched from another long-acting remained on the newly initiated treatment significantly longer than those switched from orals.

**Conclusion:**

Our results indicate the superiority of second generation long-acting antipsychotics with regard to rates of treatment discontinuation and periods of persistence to the assigned medication.

## Introduction

Schizophrenia is a chronic and severe condition that has a significant and long-lasting impact on the individual patients, their caregivers, and society. Numerous clinical trials and meta-analyses [1,2,3] have evaluated and compared the effects of oral, first generation (FGA) long acting injectable (LAI) and second generation (SGA) LAI. The contradicting outcomes of the clinical trials may result from the varying study designs but more so from the more controlled nature of the randomized clinical trials that can result in a lower than real life discontinuation rate of oral antipsychotics in clinical trial settings [1,4]. While discontinuation of treatment has been accepted as a proxy for treatment failure [5] as it is widely perceived as the main reason behind relapse, recently questions were raised whether certain patient population may benefit from a no-treatment period. [6,7]

Although a number of real-life studies have recently been published reflecting both on different aspects of potential hazard of treatment discontinuation and comparing the effectiveness of different treatments [8–14], further pragmatic, long term and full-population-based studies are needed to help understand the impact of the condition and the differences between treatments [15].

Most of the available databases lack either a long-term follow-up or full-scale healthcare information—e.g. suicide, mortality, drug usage, indication-related drug dispensation, hospitalization, co-medication, or comorbidities. Few countries have all this information optimally in place for full-population research; examples include Sweden, Denmark, Finland, Taiwan, the Republic of Korea and Hungary.

Hungary is a country with a population of approximately 10 million inhabitants who benefit from a predominantly state-run healthcare system with one central payer [16] that funds, registers, documents, and administers healthcare related events for the full population. It is also one of the few countries where access to healthcare data for research purposes is guaranteed by law [17] and data management and analysis is performed with the help of the Department of Strategic Analysis of National Health Insurance Fund Administration (NHIF).

The current analysis was performed as part of a larger project called ATTILA, (*A**ntipsychotic* *T**rea**t**ment with* *I**njectable* *L**ong* *A**cting Antipsychotics in Hungary*) in collaboration between National Health Insurance Fund Administration (NHIF), Semmelweis University Department of Psychiatry and Psychotherapy, Janssen-Cilag Hungary Ltd., an affiliate of Janssen, a Johnson and Johnson Company and an independent data research company, Healthware Consulting Ltd., in order to obtain a population level understanding of schizophrenia and to assess the real-life effectiveness of antipsychotic treatment for schizophrenia.

Our research group conducted a study on the comparison of oral and LAI SGAs in 2013 [18]. At the time of our previous work, a long-acting (LAI) formulation of risperidone (RLAI) was the only available SGA LAI in Hungary. The results highlighted the significant superiority of RLAI over oral SGA treatments in terms of treatment discontinuation. New long-acting injectable formulations of SGAs became available since the publication of our former research data, including long-acting olanzapine and long-acting paliperidone one-month formulation (PP1M). Meanwhile, several studies have also been published, analyzing the differences between oral and LAI antipsychotics in real-life settings [8–14].

Most of these studies highlighted the superiority of LAIs in terms of burden of disease, healthcare resource utilization, even though, as mentioned above, some mixed results were also published.

Although the connection between schizophrenia and higher mortality is well known [19], due the nature of this research question, this is an area where the need for more long term full populational data is particularly important. As one of the results of our ATTILA collaboration, we published an analysis of the long term full populational impact of schizophrenia on mortality and somatic morbidity [20]. Other studies focused on the connection between mortality rate of schizophrenic patients in the context of antipsychotic treatment [21,22].

The current study aimed to carry out a full-population comparison between LAIs and oral formulations of SGAs using a follow-up period of 2 years. Healthcare and drug reimbursement systems have a substantial impact on the real-life patterns of drug usage and the differences in the reimbursement may be one of the reasons behind differences of the outcomes of different studies. As PP1M became available with reimbursement for schizophrenia treatment in Hungary on 01.01.2013, this gave us the opportunity to analyze the treatment switch characteristics and the utilization of a newly-introduced antipsychotic.

The analysis was done based on the ethical approval of Central Scientific Ethical Committee, approval no. OGYI/37427-1/ 2014 dated on 23.09.2014.

## Methods

### Data source

The study used data from the nationwide, longitudinal database of the Hungarian National Health Insurance Fund (NHIF). This database contains detailed health care service data from the whole population of Hungary. Healthcare events recorded in the NHIF database are linked to individual patients by social security number (SSN), a unique patient identifier that enables longitudinal patient pathway analysis. The database includes patient-level demographic data (date of birth, geographical region, gender, date of death) and data of all reimbursed healthcare services in an inpatient and outpatient setting and drug dispensation. Medication prescription includes data regarding diagnosis (ICD-10 code), date of dispensation, brand/generic names, dosages and pharmacological formulations, intended route of administration, number of tablets, and injections.

NHIF has a right to handle patients data based on law (*Act No*. *80/1997 on mandatory health insurance coverage)* and has a right to share it on a claim basis (based on *Act 63/2012 on the re-use of public data*). Only NHIF had direct access to patient level data, other members of the research group only had access to this data indirectly, through NHIF according to data privacy regulations of NHIF. Due to this and to the retrospective nature of the study there was no need for patient level consent to the analysis.

The current comparative analysis focused on patients who were initiated on a new SGA treatment in the inclusion period between the 01.01.2012 and 31.12.2013 and were subsequently observed for a period of 2 years, up until 31.12.2015.

Owing to the availability of long-term longitudinal data which are available for analysis dating back to 2006, we were able to stratify the patients based on their diagnosis and treatment history in terms of time since diagnosis, comorbidities and previous hospitalization. Data availability from the retrospective period also enabled the comparison of treatment discontinuation of two LAI-treated patient groups: those who were switched from another SGA LAI treatment and those who were switched from an oral SGA to an SGA LAI. Recent studies [23,24,25] raised the awareness of the importance of switching patterns, maintenance treatment and to the potential benefit of switching to LAI even for patients who have been stabilized on oral treatment, but LAI-LAI switch is rarely researched to date.

Comparison of LAI groups who switched from oral treatment and those switched from another LAI enabled us to examine the importance of long-term treatment stability, and to investigate whether prior exposure to LAI treatment may be associated with a better adherence to subsequent LAIs.

### Patient selection, classification, and study periods

All SGAs reported in this study were registered, reimbursed and actively used antipsychotics in Hungary at the time of the study. The study cohort consisted of all patients who were newly prescribed and dispensed any of the following SGAs in monotherapy between the 01.01.2012 and the 31.12.2013 (inclusion period): oral amisulpride (AMIS), oral aripiprazole (ARIP), oral clozapine (CLOZ), oral olanzapine (OLAN), oral quetiapine (QUET), oral risperidone (RISP), oral paliperidone (PALI), oral ziprasidone (ZIPR), risperidone long action injection (RLAI), olanzapine long acting injection (OLAI), paliperidone long acting injection 1 monthly (PP1M). The only reimbursed SGA that could not be included in the analysis was sertindole, due to the low number of patients (n = 13 during the whole inclusion period).

**Fig 1:**
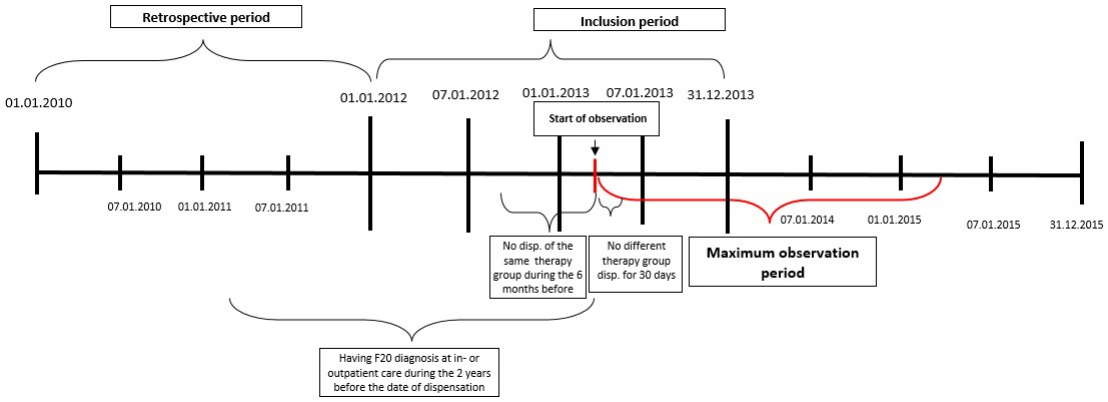
Patient inclusion and study design.

Inclusion criteria were: At least one documented schizophrenia diagnosis (F20.0—F20.9), according to ICD-10 at in- or outpatient care or drug dispensation between 01.01.2006 and 01.01.2012. Date of the first dispensation of a new antipsychotic during the inclusion period was used as the index date. Monotherapy was defined as no dispensation of any other first- or second-generation antipsychotics in the first 30 days after the index date. (See Fig 1). An exception from this definition of monotherapy was allowed for the temporary oral supplementation with risperidone at the initiation of RLAI, following the dosing recommendations in the *Summary of Product Characteristics* (SmPC) of RLAI [26]. Newly initiated therapy was defined as no dispensation of the same active ingredient and formulation (oral or LAI) for 6 months before the index date. Additional analyses were performed to compare the LAI group comprising those patients who were previously treated with another LAI with the LAI group comprising those who were switched from oral treatment or no treatment. Lack of previous LAI treatment was defined as no other LAI dispensation within 60 days before the index date.

All patients in Hungary who met the inclusion criteria were investigated in the analysis.

### Study design

The primary endpoint of this study was the all-cause 1 –and 2 year discontinuation of the initiated (“new”) antipsychotic medication. In this manuscript we focus on the primary endpoint and examine the likelihood of all cause-discontinuation as well as the median time elapsed until the all-cause discontinuation. Patients were considered to be discontinued from their respective medication if it was not dispensed after their estimated end date of exposure plus a grace period of 60 days (base case). A sensitivity analysis on the time to treatment discontinuation was carried out by applying different grace period lengths (30 and 90 days, see S1 Tables). The day of the first dispensation was considered to be the first day of exposure. Information about medication whilst in hospital was not available. If new treatment was started right after hospital discharge, then we considered it as being started during hospital stay and days in hospital were considered as part of the treatment days.

The date of discontinuation was estimated as 30 days after the day of last dispensation for the given drug, unless another antipsychotic was prescribed earlier. Patients were censored in case of death during the observation period, or after 2 years of follow-up. (2x365 days after day 1).

### Statistical analysis

The analysis focused on pairwise comparisons among the SGA medications. If a patient received concomitant antipsychotic medication after the index date, the initial treatment assignment was retained until the end of the follow up period.

Pairwise comparisons with Cox proportional-hazards regression models were used for the investigation of the likelihood of treatment discontinuation in the 2-year follow-up period. The hazard ratios with 95% confidence intervals for treatment discontinuation were determined based on Cox models. Kaplan-Meier survival analysis in the predefined monotherapy groups was used to derive the estimates of median survival times until all-cause discontinuation. Additionally, 1- and 2-year survival probabilities were estimated from the Kaplan-Meier survival analyses.

Further analysis focusing on LAI treatment history was performed. In these analyses, the Kaplan-Meier survival functions were stratified according to the previous treatment: whether patients had switched from another LAI or from oral AP in the previous 60 days. The principal aim of these analyses was to assess the importance of long-term treatment stability and to examine whether prior exposure to LAI treatments may be associated with a better adherence to subsequent LAIs (i.e. comparison of prevalent and incident users of LAI).

In order to address dissimilarities between patient characteristics in the different treatment arms, besides the estimation of the raw difference between groups, propensity score-based adjusted Cox models were fitted based on the following parameters: gender, age at baseline, previous comorbidities, number and length of previous hospitalizations and medication compliance. To compute propensity scores, the probability of receiving a certain drug over the other compared pair was estimated using a logistic regression model.

Propensity models were based on the retrospective years prior to the starting date of the therapies. When adjusting the data, the set of patients were partitioned into 5 propensity-subgroups based on the propensity score quintiles.

Reported results represent the ‘raw’ and the ‘adjusted estimates’, i.e., the propensity score and covariate-based estimates of risk (hazard) ratios along with the p-values from the statistical tests (significance level was assessed with reference to an *a priori* set α level of 0.05.

In order to evaluate the success of propensity adjustments, several conditions need to be met. If the majority of these conditions are fulfilled, the process can be considered successful. If too many conditions are violated, then there is no guarantee that the propensity approach is able to reduce the bias in the dataset. The applied scale goes from 1 (propensity accepted) to 4 (propensity failed) [27,28].

All statistical tests were performed using R software. Regarding detailed data of propensity modeling, please see S1 Tables.

## Results

During the inclusion period between 01.01.2012 and 31.12.2013 a total of 126,463 patients were prescribed antipsychotic treatment, including both first and second-generation antipsychotics. Out of these, 12,232 patients met the inclusion criteria for newly started monotherapy with SGA treatment as specified above.

The number of patients in the respective treatment arms was: 736 on AMIS, 921 on ARIP 713 on CLOZ, 2650 on OLAN, 2229 on QUET, 1614 patient on RISP, 869 on RLAI, 164 on ZIPR, 393 on OLAI, 599 on PALI, and 844 on PP1M. The average age of the patient groups ranged between 43.7 years (OLAI and ARIP groups) and 53.6 years (in the QUET and RIS groups). The percentage of younger patients (20–40 years) varied between 22% (RISP) and 42% (OLAI). The proportion of male compared to female patients was consistently lower in all treatment groups with the lower end of 34% in QUET group and the higher end of 48% in the OLAI group (Table 1).

**Table 1:** Patient characteristics at baseline.

	AMIS		ARIP		CLOZ		OLAN		QUET		RISP		RLAI		ZIPR		OLAI		PALI		PP1M	
total population (N)	N = 736		N = 921		N = 713		N = 2650		N = 2229		N = 2096		N = 869		N = 164		N = 393		N = 599		N = 844	
**1. Gender**	**n**	**%**	**n**	**%**	**n**	**%**	**n**	**%**	**n**	**%**	**n**	**%**	**n**	**%**	**n**	**%**	**n**	**%**	**n**	**%**	**n**	**%**
**male**	**281**	**38%**	**348**	**38%**	**314**	**44%**	**1160**	**44%**	**767**	**34%**	**835**	**40%**	**414**	**48%**	**61**	**37%**	**187**	**48%**	**230**	**38%**	**357**	**42%**
**2. Age at baseline**	**mean**	**SD**	**mean**	**SD**	**mean**	**SD**	**mean**	**SD**	**mean**	**SD**	**mean**	**SD**	**mean**	**SD**	**mean**	**SD**	**mean**	**SD**	**mean**	**SD**	**mean**	**SD**
**age at baseline (years),** **mean (SD)**	**49,3**	**15,0**	**43,7**	**13,7**	**49,2**	**15,5**	**47,9**	**14,7**	**53,6**	**17,4**	**52,1**	**17,0**	**46,7**	**14,6**	**46,8**	**14,2**	**43,7**	**13,6**	**46,5**	**13,4**	**47,2**	**13,0**
	**median**	**perc.**	**median**	**perc.**	**median**	**perc.**	**median**	**perc.**	**median**	**perc.**	**median**	**perc.**	**median**	**perc.**	**median**	**perc.**	**median**	**perc.**	**median**	**perc.**	**median**	**perc.**
**age at baseline (years),** **median (25–75 percentile)**	**50**	**38–60**	**42**	**33–54**	**50**	**37–60**	**49**	**37–58**	**54**	**40–66**	**53**	**39–64**	**46**	**35–58**	**46,5**	**36–56**	**43**	**32–54**	**46**	**36–56**	**47**	**38–57**
	**n**	**%**	**n**	**%**	**n**	**%**	**n**	**%**	**n**	**%**	**n**	**%**	**n**	**%**	**n**	**%**	**n**	**%**	**n**	**%**	**n**	**%**
**Age = 0–19**			**20**	**2%**			**30**	**1%**	**16**	**1%**	**29**	**1%**										
**Age = 20–29**	**68**	**9%**	**127**	**14%**	**67**	**9%**	**287**	**11%**	**185**	**8%**	**191**	**9%**	**115**	**13%**	**16**	**10%**	**67**	**17%**	**63**	**11%**	**82**	**10%**
**Age = 30–39**	**141**	**19%**	**252**	**27%**	**145**	**20%**	**514**	**19%**	**334**	**15%**	**333**	**16%**	**196**	**23%**	**34**	**21%**	**99**	**25%**	**137**	**23%**	**173**	**20%**
**Age = 40–49**	**151**	**21%**	**194**	**21%**	**126**	**18%**	**551**	**21%**	**386**	**17%**	**344**	**16%**	**181**	**21%**	**38**	**23%**	**90**	**23%**	**136**	**23%**	**216**	**26%**
**Age = 50–59**	**184**	**25%**	**216**	**23%**	**179**	**25%**	**701**	**26%**	**522**	**23%**	**511**	**24%**	**190**	**22%**	**48**	**29%**	**74**	**19%**	**161**	**27%**	**212**	**25%**
**Age = 60–69**	**120**	**16%**	**82**	**9%**	**122**	**17%**	**365**	**14%**	**346**	**16%**	**354**	**17%**	**132**	**15%**	**15**	**9%**	**52**	**13%**	**71**	**12%**	**129**	**15%**
**Age = 70–79**	**49**	**7%**	**23**	**2%**	**44**	**6%**	**152**	**6%**	**235**	**11%**	**196**	**9%**	**46**	**5%**					**24**	**4%**	**25**	**3%**
**Age = 80–89**	**13**	**2%**			**19**	**3%**	**46**	**2%**	**186**	**8%**	**123**	**6%**										
**3. Time since diagnosis** ^a^	**mean**	**SD**	**mean**	**SD**	**mean**	**SD**	**mean**	**SD**	**mean**	**SD**	**mean**	**SD**	**mean**	**SD**	**mean**	**SD**	**mean**	**SD**	**mean**	**SD**	**mean**	**SD**
**no. of months since 1st recorded** **F20 diagnosis—Mean(SD)**	**64,13**	**31,98**	**59,31**	**32,89**	**70,28**	**28,23**	**58,70**	**34,20**	**51,88**	**35,66**	**54,71**	**35,56**	**58,83**	**34,84**	**66,88**	**28,99**	**61,92**	**35,08**	**63,09**	**32,15**	**76,67**	**30,33**
	**n**	**%**	**n**	**%**	**n**	**%**	**n**	**%**	**n**	**%**	**n**	**%**	**n**	**%**	**n**	**%**	**n**	**%**	**n**	**%**	**n**	**%**
**n (%) patients with < 6 months**	**58**	**7,88%**	**87**	**9,45%**	**38**	**5,33%**	**337**	**12,72%**	**393**	**17,63%**	**337**	**16,08%**	**129**	**14,84%**			**52**	**13,23%**	**54**	**9,02%**	**42**	**4,98%**
**n (%) patients with 6-<12 months**	**23**	**3,13%**	**36**	**3,91%**	**13**	**1,82%**	**98**	**3,70%**	**78**	**3,50%**	**90**	**4,29%**	**32**	**3,68%**			**10**	**2,54%**	**19**	**3,17%**		
**n (%) patients with 12-<24 months**	**52**	**7,07%**	**80**	**8,69%**	**29**	**4,07%**	**201**	**7,58%**	**207**	**9,29%**	**175**	**8,35%**	**52**	**5,98%**	**10**	**6,10%**	**30**	**7,63%**	**39**	**6,51%**	**32**	**3,79%**
**n (%) patients with 24-<36 months**	**37**	**5,03%**	**68**	**7,38%**	**27**	**3,79%**	**160**	**6,04%**	**190**	**8,52%**	**130**	**6,20%**	**45**	**5,18%**	**13**	**7,93%**	**16**	**4,07%**	**30**	**5,01%**	**38**	**4,50%**
**n (%) patients with 36-<48 months**	**48**	**6,52%**	**52**	**5,65%**	**36**	**5,05%**	**141**	**5,32%**	**141**	**6,33%**	**114**	**5,44%**	**47**	**5,41%**			**21**	**5,34%**	**31**	**5,18%**	**34**	**4,03%**
**n (%) patients with >48 months**	**518**	**70,38%**	**598**	**64,93%**	**570**	**79,94%**	**1713**	**64,64%**	**1220**	**54,73%**	**1250**	**59,64%**	**564**	**64,90%**	**124**	**75,61%**	**264**	**67,18%**	**426**	**71,12%**	**689**	**81,64%**

^a^ time since first diagnosis appearance in the database—does not necessarily indicate the first diagnosis ever, as date of first diagnosis is not recorded; data before 2002 were not available

### All-cause discontinuation

We estimated both the 1 year and 2-year discontinuation using Kaplan-Meier survival analysis. The likelihood of treatment continuation for patients receiving LAI treatments was consistently higher than for those receiving oral treatments. The 1-year continuation with oral AP treatments varied from 17% (RISP) to 31% (OLAN) while the analogous data for LAI were between 32%(RLAI) and 64% (PP1M). The 2-year survival continuation rates were between 10–22% and 17–52%, respectively (Table 2).

**Table 2:** Proportion of patients continuing newly initiated antipsychotic initiation during the 2-year follow-up period^a^.

Treatment	AMIS	ARIP	CLOZ	OLAN	OLAI	PALI	PP1M	QUET	RISP	RLAI	ZIPR
observation time elapsed (days)^b^	n = 736	n = 921	n = 713	n = 2650	n = 393	n = 599	n = 844	n = 2229	n = 2096	n = 869	n = 164
**181**	**0.32**	**0.39**	**0.28**	**0.43**	**0.58**	**0.39**	**0.76**	**0.35**	**0.27**	**0.49**	**0.28**
**361**	**0.20**	**0.27**	**0.21**	**0.31**	**0.44**	**0.28**	**0.64**	**0.24**	**0.17**	**0.32**	**0.18**
**541**	**0.15**	**0.20**	**0.16**	**0.26**	**0.38**	**0.22**	**0.57**	**0.18**	**0.12**	**0.21**	**0.13**
**721**	**0.11**	**0.16**	**0.13**	**0.22**	**0.35**	**0.19**	**0.52**	**0.14**	**0.10**	**0.17**	**0.10**

^a^ Estimates for the proportion of the patients on the initially assigned medication at each time point are based on the non-parametric Kaplan Meier approach.

^b^ Time elapsed from the initiation of new treatment during the 2-year follow-up period

Besides the likelihood of discontinuation, treatment discontinuation was evaluated in terms of the median time elapsed from the start of a new SGA until its discontinuation. It was found that oral APs were discontinued significantly earlier than LAIs. The median time to discontinuation varied in the oral group from 57 days (CLOZ) to 121 days (OLAN). The median time to discontinuation of LAIs was significantly longer: 176 days (RLAI), 287 days (OLAI); and in the case of PP1M, the discontinuation rate did not reach the median value during the observation period (Table 3).

**Table 3:** Median time of days elapsed to all-cause discontinuation since start of the treatment^a^.

Group	Median time to discontinuation (days)
CLOZ	**57**
RISP	**58**
ZIPR	**59**
AMIS	**70**
QUET	**86**
ARIP	**91**
PALI	**103**
OLAN	**121**
RLAI	**176**
OLAI	**287**
PP1M	**not reached** ^b^

^**a**^ Median time to all-cause discontinuation is based on the non-parametric Kaplan-Meier approach

^b^ more than 50% of patients stayed on the treatment within the observation period (2 years)

### Pairwise comparisons for all-cause-discontinuation

We conducted both raw and adjusted pairwise analyses, comparing each treatment with all the others. As the focus of this study was the effectiveness of LAI treatments, we used each LAI as a basis of comparison with all other treatments in a Cox regression model. The hazard ratio (HR) for treatment discontinuation on the initially assigned treatment as compared for each of the LAI treatments was tested using unadjusted and adjusted analyses (Table 4).

**Table 4:** Raw (unadjusted) and propensity score-adjusted pairwise comparisons of LAIs and oral treatments.

**Raw hazard ratio**^a^	**PP1M** **(n = 844)**	**RLAI** **(n = 869)**	**OLAI** **(n = 393)**	**AMIS** **(n = 736)**	**ARIP** **(n = 921)**	**CLOZ** **(n = 721)**	**OLAN** **(n = 2650)**	**PALI** **(n = 599)**	**QUET** **(n = 2229)**	**RISP** **(n = 2096)**
**PP1M** **(n = 844)**	**-**	**2.65**	**1.67**	**3.61**	**3.03**	**3.58**	**2.48**	**2.76**	**3.15**	**3.87**
		**(2.343–2.997)**	**(1.427–1.954)**	**(3.18–4.097)**	**(2.683–3.42)**	**(3.151–4.067)**	**(2.229–2.761)**	**(2.412–3.15)**	**(2.827–3.511)**	**(3.47–4.319)**
		**p<0.001**	**p<0.001**	**p<0.001**	**p<0.001**	**p<0.001**	**p<0.001**	**p<0.001**	**p<0.001**	**p<0.001**
**RLAI** **(n = 869)**	**0.38**	**-**	**0.65**	**1.45**	**1.21**	**1.48**	**1.02**	**1.10**	**1.28**	**1.59**
	**(0.333–0.426)**		**(0.559–0.745)**	**(1.3–1.609)**	**(1.097–1.344)**	**(1.325–1.644)**	**(0.935–1.109)**	**(0.978–1.233)**	**(1.178–1.399)**	**(1.461–1.737)**
	**p<0.001**		**p<0.001**	**p<0.001**	**p<0.001**	**p<0.001**	**p = 0.663**	**p = 0.111**	**p<0.001**	**p<0.001**
**OLAI** **(n = 393)**	**0.60**	**1.55**	**-**	**2.14**	**1.82**	**2.16**	**1.52**	**1.65**	**1.91**	**2.34**
	**(0.511–0.7)**	**(1.34–1.786)**		**(1.85–2.479)**	**(1.579–2.099)**	**(1.863–2.501)**	**(1.335–1.733)**	**(1.418–1.923)**	**(1.676–2.181)**	**(2.052–2.671)**
	**p<0.001**	**p<0.001**		**p<0.001**	**p<0.001**	**p<0.001**	**p<0.001**	**p<0.001**	**p<0.001**	**p<0.001**
**Adjusted hazard ratio**^b^	**PP1M** **(n = 844)**	**RLAI** **(n = 869)**	**OLAI** **(n = 393)**	**AMIS** **(n = 736)**	**ARIP** **(n = 921)**	**CLOZ** **(n = 721)**	**OLAN** **(n = 2650)**	**PALI** **(n = 599)**	**QUET** **(n = 2229)**	**RISP** **(n = 2096)**
**PP1M** **(n = 844)**	**-**	**1.92**	**1.20**	**2.32**	**2.06**	**2.17**	**1.73**	**2.09**	**2.17**	**3.08**
		**(1.639–2.254)**	**(0.985–1.456)**	**(1.966–2.726)**	**(1.753–2.419)**	**(1.813–2.6)**	**(1.512–1.972)**	**(1.77–2.478)**	**(1.877–2.496)**	**(2.719–3.493)**
		**p<0.001**	**p = 0.070**	**p<0.001**	**p<0.001**	**p<0.001**	**p<0.001**	**p<0.001**	**p<0.001**	**p<0.001**
**RLAI** **(n = 869)**	**0.52**	**-**	**0.69**	**1.27**	**1.20**	**1.16**	**0.94**	**1.06**	**1.12**	**1.44**
	**(0.443–0.609)**		**(0.588–0.819)**	**(1.129–1.439)**	**(1.068–1.356)**	**(1.008–1.339)**	**(0.852–1.026)**	**(0.928–1.206)**	**(1.014–1.23)**	**(1.313–1.587)**
	**p<0.001**		**p<0.001**	**p<0.001**	**p = 0.002**	**p = 0.038**	**p = 0.158**	**p = 0.392**	**p = 0.024**	**p<0.001**
**OLAI** **(n = 393)**	**0.83**	**1.44**	**-**	**1.89**	**1.71**	**1.70**	**1.35**	**1.78**	**1.57**	**2.02**
	**(0.686–1.014)**	**(1.22–1.699)**		**(1.591–2.247)**	**(1.454–2.006)**	**(1.427–2.025)**	**(1.169–1.552)**	**(1.486–2.129)**	**(1.362–1.814)**	**(1.739–2.335)**
	**p = 0.070**	**p<0.001**		**p<0.001**	**p<0.001**	**p<0.001**	**p<0.001**	**p<0.001**	**p<0.001**	**p<0.001**

^a^ higher value means higher probability of staying on treatment represented in the row headers (LAIs)

^b^ propensity score adjustments—for details see S1 Tables.

Based on the unadjusted analyses, LAIs performed significantly better than oral APs in terms of HRs for discontinuation as follows: PP1M and OLAI were significantly better than all oral APs while RLAI was better than most of the oral APs, although this difference was not significant versus oral olanzapine (OLAN) (HR = 1.02, p = 0.663) and paliperidone oral (PALI) (HR = 1.2, p = 0.111). In the adjusted analysis, the results were as follows: PP1M and OLAI were significantly better than all oral APs, and the difference between RLAI and oral APs were significant with the exception of OLAN and PALI.

The differences between LAIs were as follows: in the unadjusted analyses, PP1M was associated with a significantly lower likelihood of all-cause discontinuation than OLAI, while in the adjusted setting this difference was not significant. PP1M and OLAI performed significantly better than RLAI in both the adjusted and unadjusted analyses.

### Impact of prior treatment on treatment discontinuation with LAIs

Significant differences were identified between the three available LAI treatments in terms of 1- and 2-year retention rates. Since from a relapse prevention perspective the continuation of treatment is of utmost importance regardless of the actual medication, it is essential to gain additional insights into the long-term continuation of antipsychotic treatment. Recent studies (29,30) also raised the attention to the economic perspective of oral-LAI switches but no publications were identified specifically on the impact of LAI-LAI switch. Thus, we conducted further analyses to identify whether a difference can be detected across the different LAI groups based on the previous treatment.

The results in Table 5 indicate that patients who were switched from a prior LAI treatment to another LAI achieved significantly longer time on treatment than patients who were either switched from oral treatment or from no previous treatment.

**Table 5:** Estimates of median time and the lower and upper quartiles of days elapsed to treatment discontinuations in different treatment groups as a function of prior treatment.

Treatment arm	Patient group based on previous treatment	25th percentile	Median	75th percentile
**RLAI (n = 858)**	no Previous LAI ^a^	58	**172**	464
**OLAI (n = 380)**	no Previous LAI	79	**287**	not reached
**PP1M (n = 507)**	no Previous LAI	123	**503**	not reached
**RLAI (n = 11)**	Previous LAI treatment^b^	174	**373**	not reached
**OLAI (n = 13)**	Previous LAI treatment	116	**not reached**	not reached
**PP1M (n = 337)**	Previous LAI treatment	351	**not reached**	not reached
**RLAI (n = 869)**	Total ^c^	58	**176**	470
**OLAI (n = 393)**	Total	79	**287**	not reached
**PP1M (n = 844)**	Total	192	**not reached**	not reached

^a^ NO PREVIOUS LAI means the patient was not treated within 60 days with any other available LAI treatment before starting on the respective arm

^b^PREVIOUS LAI treatment means the patient was treated within 60 days with any other available LAI treatment before starting on the respective arm

^c^TOTAL means all patients were considered in the respective arm, independently from their previous treatment

The median time to discontinuation in patients who were not previously treated with LAI ranged from 172 days (RLAI) to 503 days (PP1M), while the analogous estimate for patients in the RLAI group who were switched from another LAI (PP1M or LAI) was 373 days. In the respective PP1M and OLAI groups, the fraction of patients who were switched from another LAI remained less than 50% during the entire study, hence the median time could not be determined during the observation period (Table 5).

Since PP1M was a newly introduced LAI at baseline, the number of patients switched to PP1M from another LAI was relatively high: 337 or 40% of the patients switched from another LAI out of 844 patients treated with PP1M. In contrast, the patients switched to RLAI and OLAI from another LAI is low (1.2% or 11 out of 869 and 3.3% or 13 out of 393, respectively). A complete summary on the number of patients and survival rates by day on treatment is provided in S1 Tables, which also includes results of sensitivity analyses for 30-, 90- and 120-day grace periods.

**Fig 2:**
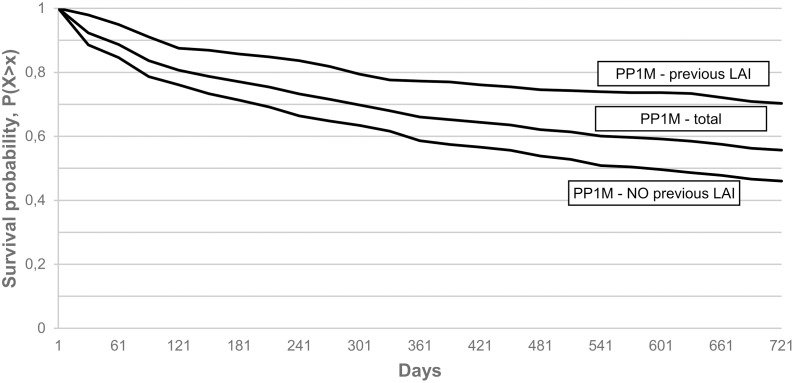
Kaplan-Meier survival of PP1M treated subgroups with 60 days grace period.

Rates of 1-year continuation in the various PP1M subgroups were: 0.56 (CI = 0.52–0.61) in the PP1M, non-previous LAI treated group, 0.64 (CI = 0.61–0.67) in the total PP1M group and 0.75 (CI = 0.70–0.80) in the PP1M, previously LAI treated groups, while 2-year continuation rates were 0.42 (CI = 0.38–0.47). 0.52 (CI = 0.49–0.55) and 0.66 (CI = 0.61-0.71) in the respective groups. (Fig 2).

## Discussion

The uniqueness of the current study is based on its full-population scale, the large number of patients (more than 12,000 newly treated schizophrenia patients in the study period), the long-term retrospective follow-up period (data available for 10 years), the prospective follow-up of the different treatment arms (2 years) and the information about treatments prior to the inclusion in the study. The primary endpoint of the current analysis was 1- and 2-year discontinuation rates of oral and LAI SGA treatments in both raw and matched estimates between the different treatment arms, with the statistical endpoint of the hazard ratio of discontinuation. Significant superiority of LAIs versus oral treatments was observed in the majority of cases, although a few treatment arms did not obtain statistical significance, as shown in the Results section.

The results are similar to other real word data in terms of time to discontinuation of both LAIs and oral treatments, even though the median time to all cause discontinuation in clinical trial settings may be different, especially in case of oral treatments. [23] The 2-year follow up further confirms and highlights even more the superiority of LAIs than the 1-year follow-up.

Little data have been published on the impact of oral-LAI switch [29] and we found no analysis of LAI-LAI switches. We found a difference in time to discontinuation between the LAI treated groups where the patients were switched to a LAI from another LAI and the groups where patients were switched from oral treatment or no treatment. This finding highlights the necessity of staying on treatment for as long as possible. As PP1M was a newly available treatment at the beginning of the study period, this gave us an opportunity to investigate the uptake of a new drug on the market. For PP1M, almost 40% of the patients were switched from another LAI, while for RLAI and OLAI the proportion of patients switched from other LAI was very small (2–3%). The trend in case of these two LAIs were, however, very similar to the findings of the PP1M groups, i.e., the median time until discontinuation is longer in patients switched from LAI than from the oral-switched group.

Consistent with our results, a large real-world study based on nationwide data from the Finnish health care register with a follow-up time of up 20 years indicate that LAIs were associated with the lowest risk of all-cause and psychiatric hospitalization among chronic and first-episode patients with schizophrenia [8]. However, unlike our study, this study also indicated a superior performance for clozapine in terms of preventing psychiatric and all-cause hospitalization. In particular, the Cox regression-based hazard ratio estimate for rehospitalization for chronic and first episode schizophrenia patients was essentially identical between clozapine and LAIs, with no significant difference between LAIs. The difference with respect to clozapine’s performance in different healthcare systems needs further explanations and studies.

An additional factor contributing to the better outcome observed after switching from LAI to LAI treatment as compared to switching from oral to LAI treatment may be patient’s preference for their current drug formulation: an earlier study reported an association between preference for drug formulation and treatment sequence whereby patients more frequently indicated that they would rather continue the current formulation than go back to the previous one [30].

Our study has its own limitations. A potential disadvantage of real-world studies compared to randomized clinical trials is the lack of randomization of patients, thus the potential lack of comparability between different patient groups. We addressed this issue by conducting both raw and adjusted pairwise analyses when comparing the individual treatments with each other. As the data are from a claims database, it relates to the reimbursement system that may have a selection bias of different treatments, e.g. newly diagnosed patients must be started with oral treatment first and can only be switched to LAIs in case of failure on orals. However, this rule has to be applied only to the first episode of this chronic disease therefore this bias is not likely to be considerable. Also, the exact date of start and end of treatment days cannot be precisely specified, and therefore in these cases estimations had to be used. Finally, drug usage data during hospitalizations could not be obtained from the database.

## Conclusions

Due to the increasing utilization of *S*GA LAI treatments, the evidence supporting the importance of adherence in the relapse prevention of schizophrenia is also increasing and the superiority of LAI formulations versus oral treatment in the real world is well documented. Our study provides further evidence from a full populational study for this superiority and demonstrates a strong association between the time to treatment discontinuation of the current/last treatment and the drug formulation (oral versus LAI) of the previous treatment. Our findings also suggest that better relapse prevention and clinical stability could be achieved by switching from one LAI to another LAI if switching is deemed necessary. Further analyses, such as long-term patient pathways including more than one LAI treatment period are needed to reveal the impact of LAI treatment on further outcomes, including death, hospitalization, co-medication or suicide.

## Supporting information

S1 Tables Patient characteristics, sensitivity analysis, detailed data, propensity evaluation and Cox analysis. (XLSX)
